# Extension of the Advanced REACH Tool (ART) to Include Welding Fume Exposure

**DOI:** 10.3390/ijerph15102199

**Published:** 2018-10-09

**Authors:** Aduldatch Sailabaht, Fan Wang, John Cherrie

**Affiliations:** 1 Institute of Biological Chemistry, Biophysics and Bioengineering, Heriot-Watt University, Riccarton, Edinburgh EH14 4AS, UK; as224@hw.ac.uk or aduldatch.s@ubu.ac.th; 2 Faculty of Science, Ubon Ratchathani University, Ubon Ratchathani 34190, Thailand; 3 Centre of Excellence in Sustainable Building Design, Heriot-Watt University, Riccarton, Edinburgh EH14 4AS, UK; fan.wang@hw.ac.uk; 4 Institute of Occupational Medicine, Research Avenue North, Edinburgh EH14 4AP, UK

**Keywords:** ART, exposure modelling, welding, fume

## Abstract

The Advanced REACH Tool (ART) is a mechanistic higher tier model to estimate inhalation exposure to chemicals using a Bayesian approach. Currently the ART model does not include exposure to welding fumes within its applicability domain; it has only been calibrated for vapours, mists, and dusts. To extend the scope to metal fumes it is necessary to review the model structure to ensure that it is appropriate, and to calibrate the updated model using available welding fume exposure measurements. This paper provides a discussion of the key modifying factors (MFs) that should be considered to extend the ART model to include welding fume exposure. Based on our literature review, welding process type, input power level, shield gas, and welding electrodes have important impact on fume formation rates (FFRs). In addition, the convective dispersion of the fume away from the weld and the interaction of the welder with the fume plume should be incorporated into the ART model. Other aspects of the ART, such as the local ventilation, do not require modification to accommodate welding fume exposure. The ART does not include the impact of wearing personal protective equipment and so this is not included in our evaluation. Proposals are made for extending the scope of the ART to include welding processes.

## 1. Introduction

Welding is one of the commonest activities carried out in the workplace, and the most popular method for joining metal materials together [1]. Due to rapid industrial development, welding is used in many processes and fields of production, and the number of welders is growing [2], with approximately two-million people around the world who are involved in welding [3]. In the UK there are about 190,000 welders, with around 73,000 professional and skilled welders, and the remainder are semi-skilled welders who carry out welding as part of their job [4,5,6].

There are many risks to health related to welding, for example, electrical shock hazards, heat or fire risks along with risks from metal fume and gases, and ultraviolet (UV) radiation from the arc [7]. These hazards mean that, if preventive measures are not adequate, in welders a range of health problems are possible, from acute health effects such as irritation of the respiratory tract, to an increased risk of asthma, neurological damage, through to lung cancer [8]. Amani et al. [9] reported that 92% of welders suffer eye injuries from UV radiation from the arc [10,11]. It is estimated that each year there are 175 welders in Britain who die prematurely from lung cancer because of welding fume exposure [12].

The electric arc or flame vaporizes the welding electrode and/or base metal, which then condenses into submicron particles called fumes. Fumes may be suspended in the air for long periods of time and may be inhaled into the lungs [13]. At high concentrations, welding fumes may cause a health hazard [14] and steps need to be taken to control exposure. Without adequate controls, the fume concentrations may be higher than the appropriate occupational exposure limit. To ensure that control measures are adequate it is prudent to undertake a risk assessment, which involves estimation of the exposure of workers to the fumes and, where appropriate, identification of the steps required to control that exposure. Exposure can be estimated by measuring the concentration of fumes inhaled by workers in a number of specific instances and/or by using a mathematical model of exposure concentration.

Exposure models are used in risk assessments and risk management to describe the association between emissions and concentrations, and to predict the impact of risk management measures. The important point in using models is that they allow us to think about the relationship between the process and environmental factors and exposure [15]. Exposure models are developed to reconstruct historical exposures or to assess possible future exposure in investigational scenarios [16].

The recent scientific interest in occupational exposure modelling has been tied to the need to quantify exposure for risk assessment required by chemical regulations in the European Union, i.e., the Registration, Evaluation, Authorisation & Restriction of Chemicals (REACH) Regulations [16], although welding fumes are not included within the scope of REACH. The European Chemicals Agency (ECHA) has outlined a tiered method for occupational exposure modelling. Tier 1 assessments use fundamental, reasonable screening models, which have some limitations in their input parameters. Several screening model tools are available at Tier 1 of REACH, such as Stoffenmanager, the European Centre for Ecotoxicology and Toxicology of Chemicals’ Targeted Risk Assessment (ECETOC TRA) tool, and the Einfaches Maßnahmenkonzept für Gefahrstoffe Exposure Tool (EMKG-Expo-Tool) [17]. Tier 1 models are generally designed to overestimate exposure, i.e., they are “conservative”. If a Tier 1 assessment cannot demonstrate there is sufficient protection for workers, then use of a Tier 2 assessment should be considered. ECHA advises that using a Tier 2 assessments can increase accuracy and validity of assessments [17]. However, these assessments are used to evaluate explicit exposure situations, and require extensive input data in order to control uncertainty [18]. The only higher tier model for inhalation exposure assessments recognised by ECHA is the Advanced REACH Tool (ART) [17]. The ART uses a Bayesian methodology; a mechanistic model evaluates inhalation exposure and any appropriate exposure measurements can then be used to update the model estimates [19]. However, neither the current ART model nor any of the Tier 1 model tools include welding processes within their scope, and they are therefore not useful for assessing health risks for welders.

This paper reviews the structure of the ART model in relation to exposure arising from welding fume to identify changes to the model’s structure to extend the applicability domain of the ART to include these processes.

## 2. Methods

### 2.1. Search Strategy

There are a number of research studies that provide insight into the factors that influence the particulate and gaseous emissions generated by welding. We searched the scientific literature using the Scopus database for articles that provided relevant information. Search terms included ‘welding fume’ and ‘factor’ in the title, abstract or keyword. Articles with publication dates between 1 January 1969 and 1 August 2018 were selected. In total 351 articles were identified, and these were then screened (by the first author) using the title and abstract to identify those that were informative.

### 2.2. Inclusion and Exclusion Criteria

Studies were included in this review only if they related to metal welding fume and had relevant data concerning potential modifying factors related to the ART model, i.e., in relation to the generation of welding fume, fume dispersal in the workplace, localized controls or the interaction of workers with the air contaminants. Studies were excluded if they were published in a language other than English or were published in a non-peer reviewed journal or report. In total 52 articles are included in this review.

## 3. Welding and Welding Fumes

**Figure 1:**
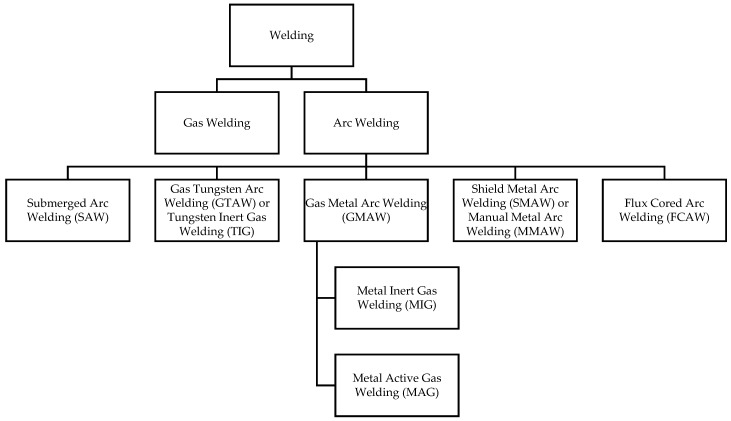
Schematic of welding methods.

Welding is a metal joining process that uses heat and/or pressure. There are allied processes of cutting, brazing, and soldering, which are often grouped along with welding. The welding process is usually classified into two main groups, i.e., gas welding and arc welding (Figure 1). In turn arc welding is categorised into two sub-groups, i.e., metal arc welding such as shielded metal arc welding (SMAW), also known as manual metal arc welding (MMAW), and gas shielded arc welding such as gas metal arc welding (GMAW), flux cored arc welding (FCAW), gas tungsten arc welding (GTAW), also known as tungsten inert gas welding (TIG), and metal inert gas (MIG) welding. Typically, the inert gases used for MIG welding are argon or helium. Metal active gas (MAG) welding is a similar process that uses a reactive gas condition, also known as ‘shielded with an active gas’ [20,21]; CO_2_ is mainly used as the shielding gas in this process. More than 90% of steel used in welding are mild steels, carbon steels or low alloy steels, with the remainder being stainless steel. Welding can also be carried out on aluminum, titanium, nickel or other metals [5].

Fumes are created when metal is heated above boiling point temperature, including heating from the arc via UV radiation, and the vapours produced rapidly oxidise and condense into a fine aerosol of solid particles [1]. The arc welding process can generate particles between 5 nm and 20 µm [22,23,24,25,26,27]. The main metal vapour elements come from the electrode [28]. Welding fume may consist of a mixture of many different metals, including aluminium (Al), chromium (Cr), hexavalent chromium (Cr(VI)), copper (Cu), iron (Fe), magnesium (Mg), manganese (Mn), nickel (Ni), phosphorus (P), tin (Sn), titanium (Ti), and zinc (Zn) [6,29,30].

In the 1970s, Heile and Hill [31] reported that the welding fume formation rate from GMAW was linked to welding conditions such as shielding gas, current, voltage, and metal transfer mode, including welding process parameters such as the electrode wire. The fume formation rate (FFR) is the rate at which welding fumes are generated; measured in mass per unit time [32]. From a review of the literature there are many factors identified that can influence the FFR, for example, electrode, shielding gas, welding parameters (voltage and current), and base metal [33]. In exposure modelling, these factors are known as modifying factors (MFs).

## 4. The ART Model

The best approach to assess exposure to hazardous substances would be to carry out personal monitoring on all workers in specific situations, but this is clearly impractical because of cost and resource limitations. Also, personal monitoring may not reflect exposure in the past because of changes in circumstances [34] and may not be representative of future conditions for similar reasons. As discussed above, many models of exposure have been developed for chemical exposure estimation to try to overcome some of these limitations. Exposure models can estimate the air concentration of hazardous substances for worker groups and periods of time for which personal monitoring is impracticable.

These models are often conceived within the source-receptor paradigm. These model inputs comprise factors related to the source of contaminant, generation, exposure control strategies and contaminant dispersal. The aims of the exposure assessment model should be to obtain accurate and precise estimates of the distribution of exposure [35]. Sometimes a model calculation can yield information more quickly than through monitoring in the workplace. Finally, models can be used to create testable hypotheses to improve the potential to evaluate actual exposures [16]. However, the validation of these model tools has not usually been performed before their release, and only sometimes afterwards [4].

Several generic exposure models exist to estimate inhalation exposure to hazardous substances [36,37]. The ART mechanistic model probably provides the most reliable generic tool for estimation of inhalation occupational exposure level. It has been selected a priori as the most suitable basis to develop a model for welding fume exposure. The ART can provide a prediction of inhalation exposure in situations where exposure data are unavailable, although it can also be used with a Bayesian updating process to incorporate available monitoring data for improved accuracy and precision [38]. The precision improvement depends on the relationship between the data and the scenario being modelled, the quantity of accessible measurements, and the variability of the measurements [39].

The ART mechanistic model uses a source-receptor structure with MFs related to the source, emission, transfer to the worker [17]. The mechanistic model, or the “prior” model in the Bayesian process, combines MFs in a multiplicative model form. Within the model, the work environment is conceptualised as two spatial volumes: the near-field (NF) surrounding the worker’s head (i.e., a cube with 1-m sides) and the far-field (FF), which comprises the reminder of the work area [40]. The ART does not include the impact of wearing personal respiratory protection on inhaled intake of contaminants.

The ART tool has some limitations because it was calibrated separately to evaluate exposure to inhalable dusts, vapours, and mists. Estimates from fumes, gases, and fibre exposures have not been available from the tool because of the lack of suitable calibration data and the lack of the appropriate model formulation for these situations [19]. To date there have been no studies reported on the development of the model for estimating these exposures, including for welding fume inhalation exposure.

## 5. Characterization of Principal Exposure Modifying Factors for Welding

Cherrie et al. [41] noted that small localized sources that involve elevated temperature, e.g., welding, present a difficult problem in terms of characterising the dispersion of the hazardous agents. For example, in these cases, the workers may place their head into, or close to, the dispersing plume, giving higher than otherwise exposures. The authors suggested further work should be considered on this issue in the ART model.

Boelter et al. [30] investigated welding fume concentrations using a two-zone mechanistic model like the ART. They used the model to estimate the FFRs in two work situations. This model is based on the breathing zone of a worker (the NF) and the surrounding area (the FF). In tests they found that the average concentration in the NF was higher than in the FF (2–10×) and was relatively independent of the general ventilation in the work areas (FF) investigated. They also identified that the general ventilation in the working area affected background concentrations and, for example, in a semi-outdoor area they found the FF concentration of welding fume was about half that in an indoor area, which was consistent with the lower measured airflow exchange rate in the latter situation. The estimated welding FFRs were similar in both situations where the field tests were carried out, but these values were approximately an order of magnitude lower than the FFRs obtained from laboratory tests. The authors recommended that the two-zone model can be used for estimation of both NF and FF air concentrations for welding fume, although it is important to have realistic data for the input parameters.

Hobson and colleagues [28] developed and validated a multivariate regression model to assess welding fume exposures using data retrieved from the published literature. They had particulate mass and manganese (Mn) concentrations from the activities of welders, which were reported as arithmetic means. Hobson et al. summarized the exposure measurements and related contextual factors, for example, sampling year, type of industry, type of welding process, ventilation type, degree of enclosure of the work environment, base metal, and sampler positioning of the worker. This study was performed to select related factors as the independent variables to build the model. The results showed that the best model included the type of welding process and degree of enclosure as the significant factors to predict the welding particulates and Mn concentrations (*r*^2^ = 0.76). The welding process in this study was identified as one of four types, i.e., SMAW, GMAW, GTAW, and FCAW and the enclosure was grouped into four categories, i.e., open space, enclosed space, confined space, and not specified space. Hobson et al. suggested that if more detailed descriptions of exposure determinants had been available this could have improved their model, for example, including electrode type as a factor in welding fume generation.

Weiss et al. [42] measured respirable and inhalable welding fumes to determine the exposure of welders to Cr and Ni. They measured both external inhalation exposure and internal exposure using biological monitoring via urine and blood. The results of this study show a strong relationship between respirable and inhalable concentrations for both Cr and Ni, with correlation coefficients (*r*) of 0.87 and 0.85, respectively. The average concentration in the inhalable welding fume was around twice the respirable welding fume concentration. In addition, the respirable Cr and Ni concentrations were significantly associated (*r* = 0.79). Likewise, Pesch et al. [43] studied the exposure to Cr(VI) and Ni in welders. They also found a strong relationship between respirable Cr and Ni concentration with *r* = 0.83, but Cr(VI) and Ni concentrations were weakly correlated (*r* = 0.42) to total welding fume concentration. In addition, the median level of exposure to welding fumes decreased from GMAW (1.06 mg/m^3^), to GTAW (0.35 mg/m^3^), and SMAW (0.25 mg/m^3^). In Weiss et al.’s paper [42], the welding process was categorised into four groups, i.e., GMAW, FCAW, GTAW, and SMAW. The average welding fume concentrations decreased, for respirable and inhalable concentrations, respectively, from FCAW (6.87 mg/m^3^ and 6.24 mg/m^3^), GMAW (1.64 mg/m^3^ and 2.71 mg/m^3^), SMAW (<0.50 mg/m^3^ and 0.82 mg/m^3^), and TIG (<0.42 mg/m^3^ and <0.58 mg/m^3^). Electrode and base metal were the most important variables that affected the concentration of Cr and Ni. Weiss et al. [42] concluded that the main factors influencing welding fume concentration and emission rate were electrode/base metal and welding process type. Pesch et al. [44] also studied the exposure to manganese (Mn) and iron (Fe) in welders. They concluded that the main factor that affected the concentration was welding process type. Moreover, Weiss et al. [42] and Pesch et al. [44] claimed that working in a confined space increased the exposure by two-fold compared to non-confined areas, and using “efficient” ventilation decreased the exposure level by approximately two-fold compared to an “inefficient” ventilation systems.

Flynn and Susi [45] developed a regression model for welding fume exposure. They obtained the welding fume exposure data for pipefitters and boiler workers from the Center for Construction Research and Training (CPWR). They fitted a regression equation to estimate Mn exposure and variance, given the total welding fume exposure. They showed that the average concentrations of Mn and total welding fume for the boiler workers were higher than for the pipefitters by three and four times, respectively. The high exposure for boiler workers was attributed to poor ventilation in the work space. There was a good relationship between manganese and total fume concentration of the pipefitters and boiler workers with *r*^2^ 0.51 and 0.64, respectively.

Generally, gas welding and arc welding need to use filler materials for welding. The filler material suitable for gas welding is provided by a non-electrode material (i.e., filler rod or filler wire) while for arc welding the electrode or base material may provide the filler. Electrodes can be divided into two types, depending on the welding process type. The first electrode type is consumed during welding providing both electrode and filling material. This electrode type is usually used for SMAW and GMAW. The second type of electrode is non-consumable and is usually used for GTAW. Therefore, the electrode types and welding process types should be considered together as factors related to welding fume generation. Due to the electrodes involvement in fume generation, the chemical composition of the electrode should also be considered in relation to the fume composition. The commonest chemical substances found in electrodes are Cr, Ni, Mn, and Fe [27], and it is important to be aware of the impact of consumables on welding fume composition.

It is clear that the location of welding activity, particularly the degree of enclosure, influences welding fume exposure. Different approaches have been taken by the various studies reviewed have to categorising ventilation and enclosure and all these seem compatible with the approach used in the ART model. Information from studies that investigated specific aspects of welding, for example, electrical current and voltage, electrode type, and shielding gas are described below.

### 5.1. Current and Voltage

The electrical current and voltage used in welding processes are probably the single greatest influencing factors on the generation of welding fumes for FCAW [46] and GMAW [47]. These factors affect the FFR due to differences in temperature of the electrode tip, type of metal transferring to the electrode, and type of current, i.e., alternating current (AC) and direct current (DC). High electrode tip temperature produces increased FFRs from an elevated evaporation rate and a rise in melting rate of the electrode, facilitating easy electrode material transfer through the arc [32].

In GMAW, the molten droplets of metal transferring from the electrode to the base material, referred to as metal transfer mode, can be categorised into five types, i.e., short-circuit transfer, globular transfer, spray transfer, pulse-spray transfer, and rotating transfer [48]. A very typical transfer mode is short-circuit transfer [49]. Each metal transfer mode has a different metal transfer stability, resulting in different FFRs. Transfer mode type depends on electrical current and voltage, for example, globular transfer operates at low current and high voltage and produces high FFRs [32]. Pires et al. [50] and Quimby and Ulrich [51] concluded that the FFR was highest for the globular transfer mode, at around 23.5 V with pulsed current and 26.5 V with steady current [51].

Hovde and Raynor [52] studied the effects of voltage on particle mass concentration in welding fume. The results showed that increasing the voltage from 16 to 21.5 V increased particle concentration, but between 21.5 and 23.5 V the fume concentrations remained almost constant.

De Meneses et al. [49] investigated short-circuit transfer mode in GMAW and concluded that increasing voltage resulted in increasing FFR; at 25 V the FFR was around five times that at 17 V. However, in their experiments it was not clear what other uncontrolled factors were important, e.g., short-circuiting current, droplets diameter, arc length, and arcing time.

For welding, the current is generally catagorised into three types, i.e., alternating current (AC), direct current electrode positive (DCEP), and direct current electrode negative (DCEN). According to Slater [32], the difference between a DCEP and DCEN may produce differences in FFRs of up to 30% in SMAW. In this process, the higher fume generation using the DCEP comes from the higher temperature of the electrode tip. Conversely, in GMAW, DCEN produced a FFR higher than DCEP [32]. This result may be related to shielding gas effects. In addition, Slater noted that use of AC in GMAW can result in fume emission similar to DCEN.

In FCAW, increasing current and input power (i.e., current × voltage) caused an increased rate of fume generation by an exponent of 1.75 and 1.19 respectively (Equations (1) and (2) below) [46].

(1) $$\mathrm{FFR}=6.2\;E-2({\mathrm{Current}}^{1.75}),\;r^{2}=0.86$$

(2) $$\mathrm{FFR}=7.1\;E-1({\mathrm{Input}\;\mathrm{Power}}^{1.19}),\;r^{2}=0.86$$

Generally, an increase in the current will also require an increase in voltage [32]. A change in voltage of 1–5% can produce up to 20% increase in the FFR [31].

In SMAW, Chan et al. [53] found that increasing the current from 90 A to 120 A resulted in increased FFR as described by the equations below:

(3) $${\mathrm{FFR}}_{\mathrm{DC}}={\left(0.0218\cdot \mathrm{Current}\right)}^{1.94}$$

(4) $${\mathrm{FFR}}_{\mathrm{AC}}={\left(0.0128\cdot \mathrm{Current}\right)}^{1.94}$$

In Equations (3) and (4), the FFR for direct current (FFR_DC_) and the FFR of the alternating current (FFR_AC_) have units of mg/min, and the current has units of A.

In GMAW, Pires et al. [54] concluded that for the same current, there is an increase of FFR with a decrease in the wire diameter, as described by the equations below:

(5) $${\mathrm{FFR}}_{0.8\;\mathrm{mm}}=0.019e^{0.0102\left(\mathrm{Current}\right)}$$

(6) $${\mathrm{FFR}}_{1.0\;\mathrm{mm}}=0.0268e^{0.0057\left(\mathrm{Current}\right)}$$

(7) $${\mathrm{FFR}}_{1.6\;\mathrm{mm}}=0.0164e^{0.0051\left(\mathrm{Current}\right)}$$

Variation in electrode diameter has a relatively small effect on the FFR [31], which is probably due to differences in welding current and voltage rather than the diameter of the electrode.

In summary, considering the current and voltage factors, it can be concluded that: the current influences FFR; increasing the current results in increasing FFR. Also, changing current also requires a change in voltage, and so these are not completely independent. It is therefore probably sufficient to just consider current as the main MF for modelling exposure to welding fume. Increasing current generally resulted in increased FFR. The type of current is important (i.e., AC or DC) and may affect FFR by around ±30%.

### 5.2. Electrode Type

For arc welding, the electrode or welding rod, is often described as the “consumable”; it is the major source of the welding fume [5,55]. Electrodes are usually of similar composition to the base material being welded. Yoon et al. [56] stated that the main fume compositions from FCAW arose from the inner flux and tubular wire more than the base metal. The most common metal used in electrodes is mild steel, although there are types of steel used that contain additional metals. For example, stainless steel electrodes may contain up to 26% Cr and 21% Ni, high-manganese hardfacing electrodes contain Mn at around 14%, and high-chromium hardfacing electrodes contain Cr up to 30% [27].

### 5.3. Shielding Gas

Shielding gas is an important factor for the quality of the GMAW, but also directly influences the FFR [47]. Several authors [31,50] stated that increasing O_2_ and CO_2_ inside the shielding gas mixture with Ar results in increasing FFR; CO_2_, especially, had a strong influence in increasing the FFR [57]. For example, the maximum FFRs of the Ar + 25% CO_2_ is approximately two-fold higher than the FFR for Ar + 2% CO_2_ [31,50], and the FFR increased from 162 to 270 mg/min for Ar + 5% CO_2_ + 2% O_2_ and Ar + 20% CO_2_ + 2% O_2_, respectively [57]. There was an insignificant effect on the FFR when 2% O_2_ was added to Ar-CO_2_ mixtures [47,50]. For the He-based mixtures, there was an insignificant change in the FFR with CO_2_ and O_2_ additions [47].

For spray transfer mode at low arc voltages in GMAW, a small increase in CO_2_ inside the shielding gas mixture with Ar and He resulted in increased FFR [27]. Moreover, Heile and Hill [31] investigated the effect of shielding gas composition (Ar + 5% O_2_ versus CO_2_) on the relationship between the FFR and current and voltage in GMAW. The relationship differed for the two gas mixtures. For the CO_2_ mixture, the FFR increased monotonically with both current and voltage. However, for the Ar-O_2_ mixture, the FFR decreased to a minimum and then rose again, separately for both increasing voltage and current. The lowest FFR was found for welding at 28 V and 250 A.

It may be concluded that increasing the current and CO_2_ concentration in the shielding gas causes a greater amount of fume. Moreover, the main fume compositions arise from the inner flux and tubular wire of the electrode more than the base metal.

## 6. Discussion

Exposure to welding fumes is an important occupational hygiene problem that results in exposure to a complex mixture of metal fumes and gases. Using appropriate exposure models could play a vital role in the assessment of welding fume exposure. Currently, there are no validated generic models for welding fume exposure assessment.

A suitable model could be based on a multiplicative structure involving a series of welding and generic specific MFs, in a similar way to the ART model used for chemicals. The key modifying factors specific to welding are related to FFRs and the convective airflows resulting in the dispersion of fumes from the source and the interaction of the welder with the fume plume. These aspects could be incorporated in the ART tool to extend the applicability domain; the remainder of the ART model is considered appropriate for welding fume exposure. The ART does not include an assessment of the impact of personal respiratory protective equipment on the amount of contaminant inhaled by workers and we do not plan to extend the model to include this for welders. However, this is a limitation that applies to all types of contaminant and, we consider the further effort should be undertaken to extend the ART model to accommodate this.

It is clear that the welding process type is a key determinant of FFR. Zimmer and colleagues [58] identified that FFR may vary by almost two orders of magnitude depending on process, with higher emissions from FCAW (750–2502 mg/min) compared to GMAW (36–372 mg/min). Part of the explanation for this variation in FFR is the process, but, within a process, factors such as input electrical current, type of electrode, and shielding gas composition may affect emissions. In general, higher current produces a higher FFR, although some studies [31,59] showed a minimum FFR in GMAW at 240–270 A, which may be due to the use of an Ar-based shielding gas. Hobson et al. [28] concluded that the average concentrations of Mn and total welding fume correlated with welding process type, with the concentration from GMAW being 0.45× that of SMAW, GTAW 1.20× and FCAW 1.24× the concentration from SMAW.

Shielding gas can affect the FFR in GMAW, where shielding with O_2_ affects FFRs less than shielding with CO_2_, especially, with O_2_ concentrations less than or equal 2% [47,50]. In practice, however, using O_2_ of more than 2% will have a negative effect on the welded joint and so this is unlikely to be used in industrial welding processes [60]. Increasing CO_2_ inside the shielding gas mixture will increase the FFR [27,57], that is, a shielding gas that contains 25% CO_2_ can produce a FFR that is more than twice that with a 2% CO_2_ in Ar-based mixture.

In practice, when occupational hygiene measurements are made, there is often little detail recorded about the welding process. Information about the process category may generally be found but details of the welding current or voltage, or even the shielding gas composition may be unavailable. This presents practical problems in adapting the ART model for welding because there is expected to be a lack of suitable data to calibrate a modified tool. However, because the functional relationship between these specific welding parameters and the FFR is known from controlled laboratory experiments it may still be possible to include them in the modified ART. In future studies of welding fume exposure, we recommend that investigators record more details about the process to facilitate the use of their data in exposure model development.

In welding, the base metal composition can vary widely, e.g., from mild steel to stainless steel containing Cr, Ni, and other toxic metals or metals such as Al. Base metal may determine to some extent the welding process used and the composition of the electrode. However, Mn can be used in flux coated electrodes for SMAW and FCAW, which can result in Mn fumes making up 0.2 to 10% of the total fume [5]. In this case, the concentrations of Mn in fume were significantly related to the welding consumables, but not the base metal [61].

In the mixture of metals in welding fume, there are observed associations between concentrations. A strong correlation between the concentrations of respirable Ni and Cr are described by the equations below [42]:

(8) $$\log_{10}\mathrm{Cr}=0.23+0.80\cdot \log_{10}\mathrm{Ni},\;r=0.79$$

There were also associations between metal concentrations and the concentration of total fume. Flynn and Susi [45] described a moderate correlation between the average concentrations of Mn and total welding fume of the pipefitters and boiler workers by the Equations (9) and (10), respectively:

(9) $$C_{\mathrm{Mn}}=0.03\left(C_{\mathrm{Total}}\right)-0.02,\;r^{2}=0.53$$

(10) $$C_{\mathrm{Mn}}=0.03\left(C_{\mathrm{Total}}\right)-0.12,\;r^{2}=0.69$$

On the other hand, Hobson et al. [28] estimated the average concentrations of Mn and total welding fume using the following non-linear equations:

(11) $${\mathrm{Mean}}_{\mathrm{Mn}}=\exp^{\left(-2.72+3.23\right)}$$

(12) $${\mathrm{Mean}}_{\mathrm{Total}}=\exp^{\left(-2.72\right)}$$

It may be possible to use these relationships to estimate individual metal fume concentrations in a welding ART model. However, there are limited data available to do this and this may add further uncertainties to the model estimates.

Enclosed or poorly ventilated areas have been shown to produce higher exposure to welding fume and the metal components when compared to open areas [28,42,45,62,63]. Confined spaces, i.e., small volume and/or low general ventilation rate, are already modelled within the ART, as is the efficacy of local controls. There is no indication that the current ART model for these factors is inappropriate for welding and it is suggested that they could be retained unmodified. However, this aspect should be investigated further during the calibration of the welding ART.

Slater [32] noted that the correlation between FFR and the breathing zone exposure depends on the convective dispersion of fume. The emissions from a welding source are buoyant relative to the surrounding air and the fume plume accelerates vertically upwards, dispersing laterally because of turbulent mixing with the surrounding air. In addition, any local ventilation control system will affect the flow characteristics of the fume plume, removing some of the emissions before disposal. When the welders head is positioned directly above the arc source, exposure levels are higher than when welding vertically with the head to the side [32]. Interaction between the welder and the source is likely to be one of the key MFs influencing welder exposure and this is a special characteristic of buoyant sources.

**Figure 2:**
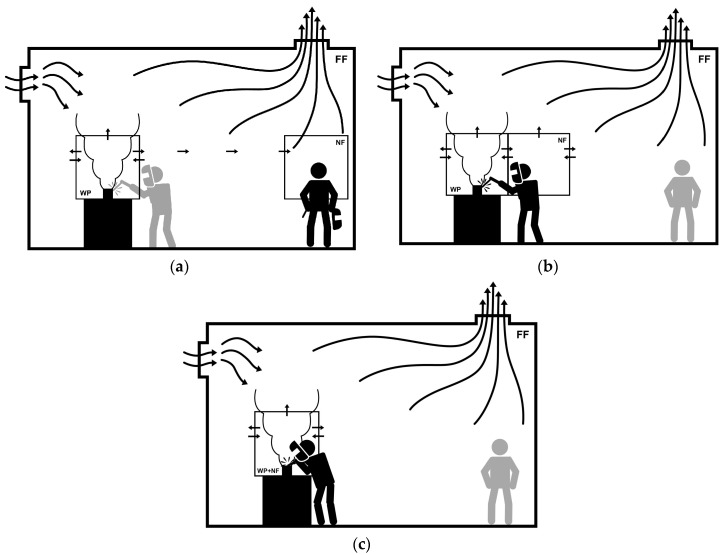
The ART model for welding fume should consider three scenarios, accounting for the interaction between the welder and the welding fume plume (WP): (**a**) Another welder colleague in a FF scheme; (**b**) A NF close to the WP scheme; (**c**) A NF and the WP are the same area scheme.

It is proposed that the ART model for welding fume should consider three scenarios, accounting for the interaction between the welder and the welding fume plume (WP). The first scenario is where the welder is more than 1m from another welding colleague and exposure to fume arise from her far-field (FF); there are no welding sources in her near-field (NF) (Figure 2a). The second scenario shows a welder close to the fume plume, but the NF is not within the plume (Figure 2b). The last scenario has a welder working with their NF within the fume plume, i.e., essentially the WP and NF spaces coincide (Figure 2c).

We propose to modify the ART algorithm to incorporate the three scenarios described above. This modification will require the estimation of the amount of time a welder in specific scenarios spends in each of these three scenarios. For exposure arising from the welders FF (Figure 2a) the form of the deterministic equation remains the same as in ART, although the MFs will differ to include welding-specific items (Equation (13)). Here the inhalable mass concentration (C_FF_) is:

(13) $$C_{\mathrm{FF}}=\left(E_{\mathrm{FF}}\cdot H_{\mathrm{FF}}\cdot {\mathrm{LC}}_{\mathrm{FF}}\cdot {\mathrm{Seg}}_{\mathrm{FF}}\right)\cdot D_{\mathrm{FF}}\cdot \mathrm{Sep}$$

where substance emission potential (E) is a multiplicative function of welding process (PRO), electrode type (ELT), and shielding gas (GAS). Activity emission potential (H) is a multiplicative function of current (CUR), and voltage (VOT). Seg represents isolation around sources and work area. D is a ventilation type, which is related to the degree of enclosure and general ventilation airflows. Sep is a personal enclosure around the worker, e.g., an air-conditioned cab.

In the welding ART model, the NF contributions are divided into those arising from time adjacent to the welding plume (C_NF−WP_) and those from time when the welder is in the welding plume (C_NF+WP_). In both these situations the MFs for substance emission potential and activity emission potential are the same, i.e., E_NF_ and H_NF_. However, for the time where her head is adjacent to the plume the local control (LC) and dispersion factors (LC_NF−WP_ and D_NF−WP_) will differ from those when her head is in the welding plume (LC_NF+WP_ and D_NF+WP_). These equations are shown below:

(14) $$C_{\mathrm{NF}-\mathrm{WP}}=E_{\mathrm{NF}}\cdot H_{\mathrm{NF}}\cdot {\mathrm{LC}}_{\mathrm{NF}-\mathrm{WP}}\cdot D_{\mathrm{NF}-\mathrm{WP}}$$

(15) $$C_{\mathrm{NF}+\mathrm{WP}}=E_{\mathrm{NF}}\cdot H_{\mathrm{NF}}\cdot {\mathrm{LC}}_{\mathrm{NF}+\mathrm{WP}}\cdot D_{\mathrm{NF}+\mathrm{WP}}$$

The total exposure (C_Total_) is calculated by combining these concentrations in a time-weighted format:

(16) $$C_{\mathrm{Total}}=\frac{1}{t_{\mathrm{Total}}}\left(C_{\mathrm{FF}}\cdot t_{\mathrm{FF}}+C_{\mathrm{NF}-\mathrm{WP}}\cdot t_{\mathrm{NF}-\mathrm{WP}}+C_{\mathrm{NF}+\mathrm{WP}}\cdot t_{\mathrm{NF}+\mathrm{WP}}+0\cdot t_{\mathrm{Non}-\exp}\right)$$

where, t_Total_ = time of a work shift, t_FF_ = exposure time for a welding source within the FF, t_NF−WP_ = exposure time when her head is adjacent to the plume, t_NF+WP_ = exposure time when her head is in the welding plume, and t_Non−exp_ = time with non-exposure:

(17) $$t_{\mathrm{Total}}=\left(t_{\mathrm{FF}}+t_{\mathrm{NF}-\mathrm{WP}}+t_{\mathrm{NF}+\mathrm{WP}}+t_{\mathrm{Non}-\exp}\right)$$

We use the data that has been obtained from literature review to assign model multipliers as described in Table 1. The multipliers are based on the fume concentration using the exposure data collated from the literature (see Appendix A). There is insufficient data to reliably assign numeric values for all the model modifying factors categories, and so we have had to use an element of judgement in these assignments. While this is not optimal it is pragmatic and in keeping with the approach used in developing the original ART. Additionally, the reliability of the final model will be evaluated in subsequent validity studies.

Although there are data in the literature to determine the parameter values for the MFs affecting FFR, there are no data to determine the MFs for the dispersion scenarios. Further research in needed to investigate the importance of these factors and to determine the numeric multiplier to be applied. 

Once the ART model has been modified to include welding specific MFs it will be necessary to calibrate the model to provide estimates of welding fume exposure for different metal components in units of mass per unit volume of air. To do this it is necessary to have access to a wide range of exposure measurements from different welding processes, in different scenarios with different patterns of work. It will therefore be necessary to compile a database of existing and/or new measurements containing the associated MFs. Once finalised the welding ART model could be incorporated into a suitable software system and could then provide a useful tool for occupational hygienists and others to help manage metal fume exposure of welders.

## 7. Conclusions

At the moment there are no generic models to assess exposure to welding fumes. The ART model provides a basis for this type of model, but it will require adaption to make it suitable. We identified that welding process type, input power level, shield gas, and welding electrodes effect fume formation rates and should be incorporated in a welding-ART model. Additionally, the modified model needs to take account of the convective dispersion mechanism for welding fume and the potential interaction of the welder with the fume plume. Other aspects of the ART model, such as local control measures, do not need to be modified. The scientific literature provides some guidance to derive the magnitude of model modifying factors, but it is also necessary to rely on expert judgement for some specific parameter values. Further research is needed to derive the numeric values for the general ventilation modifying factors in the welding-ART.

## Figures and Tables

**Table 1:** Modifying factor scoring for ART welding fume exposure model.

MFs of ART for Welding Fume Classification		Multiplier	References
Substance Emission Potential [E]			
Welding Process Type (PRO)	Arc Welding		[27,28,64]
	-Flux Core Arc Welding (FCAW)	1.0	
	-Shielded Metal Arc Welding (SMAW)	0.8	
	-Gas Metal Arc Welding (GMAW)	0.4	
	-Plasma Arc Welding (PAW)	0.1	
	-Gas Tungsten Arc Welding (GTAW)	0.03	
	-Submerged Arc Welding (SAW)	0.01	
	Gas Welding	0.4	
	Laser Beam Welding	0.02	
Welding Electrode (ELT)	Non-stainless Steel	1.0	[56]
	Stainless Steel	0.8	
Shielding Gas (GAS)	CO_2_	1.0	[47,57]
	Ar/He-based Mixture		
	-with 18% CO_2_	0.7	
	-with 12% CO_2_	0.6	
	-with 10% CO_2_	0.5	
	-with 6% CO_2_	0.5	
	-with 5% CO_2_	0.5	
Activity Emission Potential [H]			
Current (CUR)	>230 A	1.0	[46,50]
	120–230 A	0.7	
	<120 A	0.3	
Voltage (VOT)	>30 V	1.0	[46]
	22–30 V	0.7	
	<22 V	0.3	

## Appendix A. Description of the Scoring of Multipliers in Table 1

Hobson et al. [28] provided the regression coefficient for welding process types with 1.24 (FCAW), 1.00 (SMAW), 0.45 (GMAW), and −1.20 (GTAW). We convert these coefficients into the multipliers by using FCAW as the reference category (1.0). Therefore, the multipliers for each of the four welding process types are 1.00 (FCAW), 0.8 (SMAW), 0.4 (GMAW), and 0.03 (GTAW). Some papers describe SAW has having the lowest FFR. Then, in ascending order, come laser beam welding, GTAW, and PAW. The FFR of gas welding was expressed equal GMAW [64]. From this information, we assigned the multipliers as the Table 1, based on the judgement of the researchers.

Yoon et al. [56] showed that metal fumes identified higher concentrations with non-stainless steel welding (51.6%) compared with stainless steel (40.2%); based on this, the ratio between the fume content of non-stainless steel electrode and the fume contents of stainless steel electrode can be expressed as 1:0.8.

The influence of shielding gas mixtures on the FFR is a result of increasing CO_2_ [57]. The FFR results of Carpenter et al.’s study [47] were used to obtain multipliers for the shielding gas factor. The FFRs were converted to the multipliers by using a FFR of 100% for CO_2_ shielding gas (568 mg/min) as the reference. For example, the FFR of Ar-18% CO_2_ is 396 mg/min. Therefore, 396 divided by 568 equals 0.7.

Equation (1) gives the FFR in relation to electrical current. From this equation, we assigned the FFR of the current higher than 230 A (855.81 mg/min) as the reference and then calculated the FFR for other currents compare with the reference. For example, the FFR of current 120 A is 273.77 mg/min, the ratio of 273.77 to 855.81 is 0.3 that is the multiplier of the current lower than 120 A.

The current and voltage were categorised into three groups, i.e., high input power (>230 A, >30 V), optimal input power (120–230 A, 22–30 V), and low input power (<120 A, <22 V) [46]. Slater [32] reported that an increase in the current will also require an increase in voltage. This information was used to assign the multipliers for voltage in relation to current.
